# Research on Forced Vibration Model of End Effector Under Low-Frequency Excitation and Vibration-Suppression Technology

**DOI:** 10.3390/mi16020131

**Published:** 2025-01-23

**Authors:** Changqi Li, Henan Song, Ruirui Li, Jianwei Wu, Xiaobiao Shan, Jiubin Tan

**Affiliations:** 1 The Key Lab of Ultra-Precision Intelligent Instrumentation Engineering (Harbin Institute of Technology), Ministry of Industry and Information Technology, Harbin 150001, China; 18b901029@stu.hit.edu.cn (C.L.); liruirui@hit.edu.cn (R.L.); wujianwei@hit.edu.cn (J.W.); jbtan@hit.edu.cn (J.T.); 2 State Key Laboratory of Robotics and System, Harbin Institute of Technology, Harbin 150001, China; xiaobiaoshan@hit.edu.cn

**Keywords:** end effector, multi-stepwise beam, fixed-end excitation, vibration response, vibration suppression

## Abstract

The positioning accuracy of the end effector is the core index that affects the robot’s performance. However, to achieve lightweight and functional requirements, the construction of end effectors is becoming more complex. Lightweight design through slotting is becoming more common. This leads to the fact that the traditional mathematical model cannot accurately characterize the vibration of the end effector. This study proposed the multi-stepwise beam model. It employed the separation of variables and element transmitting method to obtain the mathematical model of the modal shape functions and the natural frequencies. Meanwhile, the vibration response of the end effector under fixed-end excitation was analyzed, and the conclusions were made through experimental research. The direct inverse controller was presented to achieve vibration suppression. The experimental results indicate that the amplitude suppression rate reaches 50%. The system’s equations of motion were solved numerically to analyze the exact relationships for the response and excitation of the beam considered.

## 1. Introduction

Employing the beam structures as end effectors is a general-purpose tool in the fields of aeronautics and astronautics [1], intelligent manufacturing [2], robotics [3], industrial engineering [4], and other devices for moving and driving an object precisely. With the increase in functional requirements and application scope, the structure of the cantilever beams is becoming increasingly complex. This leads to shortcomings that are tethered to the innate operating concept. The complex structure generates complex vibration characteristics that traditional mathematical beam structure vibration models can no longer describe. This paper proposed the multi-stepwise beam equivalent method to characterize the end effector and built the analytical model and the solution under fixed-end excitation for the first time.

The end effector is subjected to the robot body, leading to the fixed-end oscillation excitation. The excitation leads to unwanted vibration and causes the positioning accuracy error. The end effectors generally operate around their subsequent resonance excited by the robot body. Therefore, mastering the natural frequency and the response, like the maximum vibration amplitudes of the system when operating in the transient state, is of great importance. The traditional cantilever beam structure has been studied in detail [5,6]. However, due to the complexity and variability of the end effector section, the solution results of the traditional model are no longer accurate.

In view of the exciting research, the research idea of the dynamic model of the end effector is to obtain its natural frequency and modal function by establishing the free vibration model [7,8], normally based on the energy method [9,10,11,12] and Euler beam theory. Carpenter [13] derived finite beam elements incorporating piezoelectric materials through energy methods. Liu et al. [14] obtained the static non-uniform beam equation under external static loads. Xu et al. [15] analyzed the dynamics model of buckled MEMS beams under low-frequency vibrations. The model was established based on the Euler beam, and its subsequent research was mainly based on the perturbation method [7,16], Laplace transform [17], and the transfer matrix method. The transfer matrix method is mainly used in uneven materials and section shape changes. Yu et al. [18] explored variable-section piezoelectric laminated beams’ bending vibration transfer equations. Du et al. [9] presented the model for functionally gradient beams to discuss its characteristics; similarly, Cuma et al. [19], Rjoub et al. [20], Chen et al. [21], Wu [22], and others have contributed to this area.

When analyzing the vibration characteristics of end effectors, scholars often overlook the influence of base or fixed-end excitation [23,24]. The fixed end of the cantilever beam is treated as unmoved, and the excitation is equivalent to the inertia characteristic force at the free end. However, in practical work, the excitation at the fixed end of the cantilever beam will be coupled with its vibration characteristics due to body posture changes. However, there is still relatively little research in this area. Freundlich [25,26] proposed that the base excitation was equivalent to rigid body motion and vibration by adding mass to the free end of the cantilever beam, which is the same mindset as Li et al. [27] and Jahangiri et al. [28]. Naiera-Flores et al. [29] explored approximating nonlinear responses to harmonic base-excitation using fixed-interface quasi-static modal analysis. Mohanty et al. [30] established the dynamic model under the moving load and base excitation. Latalski et al. [31] developed the rotating thin-walled composite beam model under harmonic base excitation. Xu et al. [32] also conducted an exploration of rotational excitation. Won et al. [33] analyzed the stick-slip vibration of a cantilever beam subjected to harmonic base excitation. There is still no research on the vibration characteristics of stepwise beams and the response characteristics under base excitation.

Hence, this paper proposed an analytical solution for the end effector subjected to base motion. The mathematical analytic equation is built based on the multi-stepwise beam model and the transfer matrix method. The characteristic equation, modal frequencies, eigenfunction, and orthogonality condition are obtained through a separate variable method. The equations of motion of the system are solved numerically. The exact relationships for the response and excitation of the beam considered are derived. At the same time, based on this method, the vibration characteristics and vibration suppression techniques of end effectors were explored and applied to solve engineering problems.

## 2. Methodology

### 2.1. Formulation of the Problem

**Figure 1:**
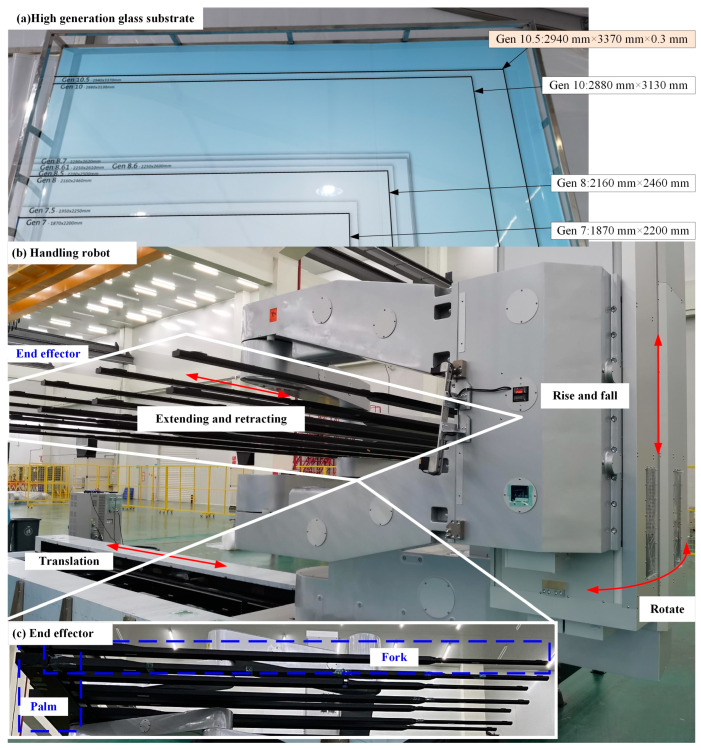
The picture of the substrate-handling robot.

With the rapid development of the display industry, the demand for substrate-handling robots has increased. High-speed, large size, and lightweight handling robots have become the focus of research as shown in Figure 1. However, due to the large size, ultra-thin structure, and fragility of the substrate, the requirements for the end effector are increased. The vibration characteristics of the end effector caused by the moving of the handling robot body need to be precisely described and controlled.

**Figure 2:**
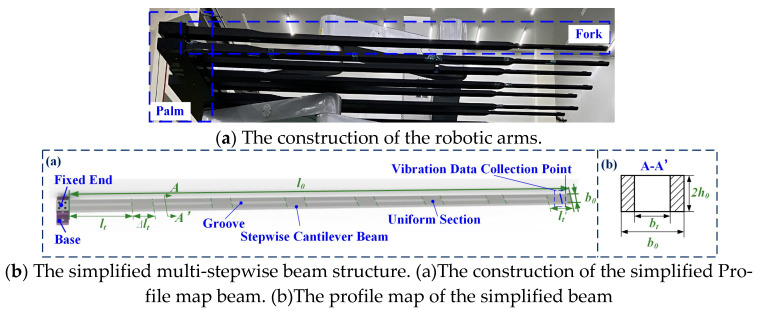
Schema of the system.

The robotic arms are shown in Figure 2a, and any single fork is simplified as the multi-stepwise beam, as shown in Figure 2b, which has both regular and irregular changes in cross-section. The parameters of the robotic arms are shown in Table 1. This form of beam is widely used in high-end equipment and industrial machinery.

### 2.2. Mathematical Model and Solution

**Figure 3:**
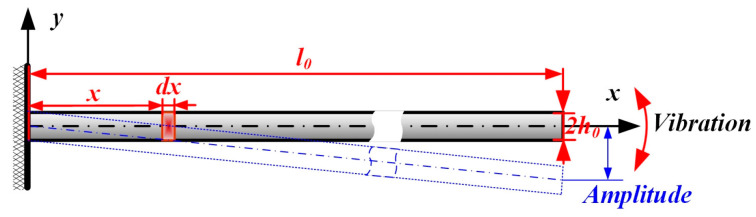
The traditional structure of the cantilever beam.

Taking an element analysis on a cantilever beam as an example, as shown in Figure 3, the beam is composed of the length of *l*_0_, the thickness of 2*h*_0_, and the width of *b*_0_. The distance between the fixed end and element *dx* is *x*. The beam structure is treated as the Bernoulli–Euler beam, meaning that the effects of shear deformation and the second moment of cross-section area with respect to the centroidal axis and the damping can be ignored when low-frequency vibration is involved.

For the homogeneous, variable cross-section cantilever beam, its vibration equation is expressed as follows:

(1) $$EI(x)\frac{\partial^{4}w\left(x,t\right)}{\partial x^{4}}+\rho S(x)\frac{\partial^{2}w\left(x,t\right)}{\partial t^{2}}=0$$

where *E* is Young’s modulus, *I*(*x*) is the second moment of area, ρ is the density of the beam, *w*(*x*,*t*) is the deflection curve, and *S(x)* is the cross-section area. Through the method of separating variables, it is obtained that $w\left(x,t\right)=Y\left(x\right)q\left(t\right)$, where *Y*(*x*) is the mode function, and *q*(*t*) is the time function obtained using the separating variables method. Hence, Equation (1) is simplified as follows:

(2) $$\begin{array}{c} EI(x)Y^{\left(4\right)}\left(x\right)-{\omega_{j}}^{2}\rho S(x)Y\left(x\right)=0\\ q^{\prime \prime }\left(t\right)+{\omega_{j}}^{2}q\left(t\right)=0 \end{array},$$

where $\omega_{j}$ is the *j*-th natural frequency.

The general solution of the mode function is expressed as follows:

(3) $$Y_{j}(x)=A_{j}\sin\beta_{j}x+B_{j}\cos\beta_{j}x+C_{j}\sin h\beta_{j}x+D_{j}\cos h\beta_{j}x$$

where $\beta_{j}=\sqrt[{4}]{\frac{\rho S(x)}{EI(x)}\omega_{j}^{2}}$, and *A*_j_, *B*_j_, *C*_j_, and *D*_j_ are the undetermined coefficients of the j-order mode function.

The boundary condition of the cantilever beam, the flexural rigidity ${\left(EI\right)}_{i}$, and the linear density ${\left(\rho S\right)}_{i}$ will not change with the change of j. Therefore, the boundary condition is expressed as follows:

(4) Y1j(0)=0,Y1j(′0)=0(EI)iYij(″l0)(EI)iYij(‴l0)=00

By incorporating the equation into the boundary conditions, the transcendental equation for the natural frequency of the cantilever beam can be inferred. Substituting the natural frequency to Equation (3) gives the solution of the model shape. By using the beam splitting method, the beam is decomposed into *n* segments with the same cross-section. The length of the *i*-th segment is $l_{i}$, and the flexural rigidity ${\left(EI\right)}_{i}$ and the linear density ${\left(\rho S\right)}_{i}$ can be expressed as follows:

(5) $${\left(EI\right)}_{i}=\frac{1}{l_{i}}\int_{x_{i-1}}^{x_{i}}EI(x)dx$$

(6) $${\left(\rho S\right)}_{i}=\frac{1}{l_{i}}\int_{x_{i-1}}^{x_{i}}\rho S(x)dx$$

According to Equation (3), the *j*-th mode function of the *i*-th beam can be expressed as follows:

(7) $$Y_{ij}(x)=A_{ij}\sin X_{i}+B_{ij}\cos X_{i}+C_{ij}\sinh X_{i}+D_{ij}\cosh X_{i},$$

where $X_{i}=\beta_{j}(x-x_{i-1})$, $\beta_{j}=\frac{{\left(\rho A\right)}_{i}}{{\left(EI\right)}_{i}}{\omega_{j}}^{2}$, $x_{i-1}\leq x\leq x$, $x_{0}=0$, and $\omega_{j}$ is the natural frequency of the cantilever beam. Owing to the continuity principle of the beam, the deflection, rotation angle, bending moment, and shear stress of the cantilever beam are all equal, while $x=x_{i}$. Hence, the mathematical expression of the *j*-th mode function is the following:

(8) $$\left[\begin{array}{c} Y_{i+1}\left(x_{i}\right)\\ Y_{i+1}^{\prime }\left(x_{i}\right)\\ {\left(EI\right)}_{i+1}Y_{i+1}^{\prime \prime }\left(x_{i}\right)\\ {\left(EI\right)}_{i+1}Y_{i+1}^{\prime \prime \prime }\left(x_{i}\right) \end{array}\right]=\left[\begin{array}{c} Y_{i}\left(x_{i}\right)\\ Y_{i}^{\prime }\left(x_{i}\right)\\ {\left(EI\right)}_{i}Y_{i}^{\prime \prime }\left(x_{i}\right)\\ {\left(EI\right)}_{i}Y_{i}^{\prime \prime \prime }\left(x_{i}\right) \end{array}\right]$$

By employing matrix $M_{i}=\left[\begin{array}{cccc} \sin X_{i} & \cos X_{i} & \sin hX_{i} & \cos hX_{i} \end{array}\right]$ and $E_{i}={\left[\begin{array}{cccc} A_{i} & B_{i} & C_{i} & D_{i} \end{array}\right]}^{T}$, Equation (8) can be expressed as $Y_{i}(x)=M_{i}\cdot E_{i}=Y_{i+1}(x)=M_{i+1}\cdot E_{i+1}$. By introducing it into Equation (8), the transfer matrix can obtain the following:

(9) $$E_{i+1}=Z_{i}\cdot E_{i}$$

Equation (9) is derived as follows:

(10) $$E_{i}=Z\cdot E_{1},Z=Z_{i-1}\cdot Z_{i-2}\cdot \ldots \cdot Z_{2}\cdot Z_{1}$$

Substituting Equation (4) into Equation (10), one can obtain the following

(11) $$\Lambda E_{i}=0,$$

where Λ is the coefficient matrix obtained from the boundary conditions. Combining Equation (10) and Equation (11), one can obtain the following:

(12) $$\Lambda ZE_{i}=\lambda E_{i}=0,$$

where λ=ΛZ. The boundary condition is the key to obtaining the parameters in Equation (11). As for the fixed end, the displacement and velocity are zero. The mathematic equations are the following:

(13) $$\begin{array}{ccc} \left\{\begin{array}{c} Y_{1}(0)=0\\ Y_{1}^{\prime }(0)=0 \end{array}\right. & or & \left\{\begin{array}{c} Y_{i}(0)=0\\ Y_{i}^{\prime }(0)=0 \end{array}\right. \end{array}$$

Equation (13) is derived into the following:

(14) $$\begin{array}{ccc} A_{1}+C_{1}=0,B_{1}+D_{1}=0 & or & A_{i}+C_{i}=0,B_{i}+D_{i}=0 \end{array}$$

From simultaneous Equations (12) and (14), we can derive the following:

(15) $$\left[\begin{array}{cccc} \lambda_{11} & \lambda_{12} & \lambda_{13} & \lambda_{14}\\ \lambda_{21} & \lambda_{22} & \lambda_{23} & \lambda_{24} \end{array}\right]{\left[\begin{array}{cccc} A_{1} & B_{1} & -A_{1} & -B_{1} \end{array}\right]}^{T}=0$$

The matrices A1 and B1 are not all zero. Therefore, the determinant of the coefficient is zero, and from Equation (15), the following can be derived:

(16) $$\left|\begin{array}{cc} \lambda_{11}-\lambda_{13} & \lambda_{12}-\lambda_{14}\\ \lambda_{21}-\lambda_{23} & \lambda_{22}-\lambda_{24} \end{array}\right|=0$$

As for the free end, the bending moment and the shear force are zero, which is derived as follows:

(17) $$\begin{array}{ccc} \left\{\begin{array}{c} {}[{\left(EI\right)}_{1}Y_{1}^{\prime \prime }(0)]\prime =0\\ {\left(EI\right)}_{1}Y_{1}^{\prime \prime }(0)=0 \end{array}\right. & or & \left\{\begin{array}{c} {}[{\left(EI\right)}_{i}Y_{i}^{\prime \prime }(0)]\prime =0\\ {\left(EI\right)}_{i}Y_{i}^{\prime \prime }(0)=0 \end{array}\right. \end{array}$$

Equation (17) is generally used to build the matrix equation. As shown in Equation (11), we obtained the following:

(18) $$\Lambda E_{i}=0,$$

where $E_{i}={\left[\begin{array}{cccc} A_{i} & B_{i} & C_{i} & D_{i} \end{array}\right]}^{T}$, Λ=−(EI)iβisin2(βil0)− (EI)iβicos2(βil0) (EI)iβisinh2(βil0) (EI)iβicosh2(βil0)−(EI)iβicos3(βil0)(EI)iβisin3(βil0)(EI)iβicosh3(βil0)(EI)iβisinh3(βil0).

Due to the repeatability of the internal structure of the end effector, the equivalent cantilever beam is divided into 14 sections, as shown in Figure 2b. The specific parameters are as follows.

(19) $$\rho S(x)=\left\{\begin{array}{c} 2h_{0}\rho b_{0},x\in \left(x_{i-1},x_{i}\right),i=2n-1,n=1,2,\ldots 7\\ 2h_{0}\rho \left(b_{0}-b_{t}\right),x\in \left(x_{i-1},x_{i}\right),i=2n,n=1,2,\ldots 6 \end{array}\right.$$

(20) EI(x)=2h0Eb0−3bt312,x∈xi−1,xi,i=2n,n=1,2,…62h0Eb0312,x∈xi−1,xi,i=2n−1,n=1,2,…7

(21) $$l_{i}=\left\{\begin{array}{c} l_{t},x\in \left(x_{i-1},x_{i}\right),i=2n-1,n=1,2,\ldots 7\\ l_{\Delta t},x\in \left(x_{i-1},x_{i}\right),i=2n,n=1,2,\ldots 6\\ l_{tt},x\in \left(x_{i-1},x_{i}\right),i=2n,n=7 \end{array}\right.$$

By substituting Equations (19)–(21) into Equation (9), the transfer matrix is derived as follows:

(22) Z1=Z3=Z5=Z7=Z9=Z11=Z13=Zt=b0 2cosα1b0−bt p1+12 b0−3bt3−b0 2sinα1b0−bt p1+12 b0−3bt3−b0 2coshα1 b0−bt p1−12 b0−3bt3−b0 2sinhα1 b0−bt p1−12 b0−3bt36 ω2 ρ sinα1b0−bt p1+1E b0−3bt36 ω2 ρ cosα1b0−bt p1+1E b0−3bt3−6 ω2 ρ sinhα1 b0−bt p1−1E b0−3bt3′−6 ω2 ρ coshα1 b0−bt p1−1E b0−3bt3−b0 2cosα1 b0−bt p1−12 b0−3bt3b0 2sinα1 b0−bt p1−12 b0−3bt3b0 2coshα1 b0−bt p1+12 b0−3bt3b0 2sinhα1b0−bt p1+12 b0−3bt3−6 ω2 ρ sinα1b0−bt p1−1E b0−3bt3−6 ω2 ρ cosα1 b0−bt p1−1E b0−3bt36 ω2 ρ sinhα1 b0−bt p1+1E b0−3bt36 ω2 ρ coshα1b0−bt p1+1E b0−3bt3,

where $\alpha_{1}=\frac{12l_{t}\omega^{2}\rho \left(b_{0}-b_{t}\right)}{E\left({b_{0}}^{3}-{b_{t}}^{3}\right)}$, $p_{1}=\frac{b_{0}{\left(b_{0}-b_{t}\right)}^{2}}{{b_{0}}^{3}-{b_{t}}^{3}}$.

(23) Z2=Z4=Z6=Z8=Z10=Z12=ZΔt=cosα2 2 b0+2bt2 b0−3bt32 b0 3b0−bt2−sinα22 b0+2bt2 b0−3bt32 b0 3b0−bt2−bt coshα2 b0−3bt3 2 b0+bt2 b0 3b0−bt2−bt sinhα2 b0−3bt3 2 b0+bt2 b0 3b0−bt26 ω2 ρ sinα2 2 b0+2bt2E b0 3b0−bt6 ω2 ρ cosα2 2 b0+2bt2E b0 3b0−bt−6 bt ω2 ρ sinhα22 b0+btE b0 3b0−bt−6 btω2 ρ coshα2 2 b0+btE b0 3b0−bt−btcosα2b0−3bt3 2 b0+bt2 b0 3b0−bt2bt sinα2b0−3bt3 2 b0+bt2 b0 3b0−bt2coshα2 2 b0+2bt2 b0−3bt32 b0 3b0−bt2sinhα2 2 b0+2bt2 b0−3bt32 b0 3b0−bt26 ω2 ρ coshα22 b0+2bt2E b0 3b0−bt−6 bt ω2 ρ cosα2 2 b0+btE b0 3b0−bt6 ω2 ρ sinhα22 b0+2bt2E b0 3b0−bt6 ω2 ρ coshα2 2 b0+2bt2E b0 3b0−bt

where $\alpha_{2}=\frac{12\Delta l_{t}\omega^{2}\rho }{E{b_{0}}^{2}}$.

Rewriting Equation (10) obtains the transfer matrix as follows:

(24) $$Z_{14}=Z_{13}Z_{12}Z_{11}Z_{10}Z_{9}Z_{8}Z_{7}Z_{6}Z_{5}Z_{4}Z_{3}Z_{2}Z_{1}$$

By substituting Equations (22)–(24) into Equation (12), one can obtain the following:

(25) $$\lambda =\Lambda Z=\Lambda Z_{14}=\Lambda (Z_{t}Z_{\Delta t}{)}^{6}Z_{t}$$

**Theorem** **1.** *For any matrix A, B, there is* $\left|\mathrm{AB}\right|=\left|A\right|\times \left|B\right|$ *. Hence, the determinant of Equation (25) is redrafted as follows:*

(26) $$\left|\lambda \right|=\left|\Lambda \right|\times {\left(\left|Z_{t}\right|\times \left|Z_{\Delta t}\right|\right)}^{6}\times \left|Z_{t}\right|$$

In Equation (26), the matrix Λ is a non-square matrix. Hence, we proposed Lemma 1 to solve the problem of solving the non-square determinant.

**Lemma** **1.** *For any non-square matrix* $B_{n\times k}=\left[\begin{array}{ccc} b_{11} & ... & b_{1k}\\ ... & ... & ...\\ b_{n1} & ... & b_{nk} \end{array}\right]$*, when n > k, there is the following: *$|B_{n\times k}|=\sqrt{\left|{B_{n\times k}}^{T}B_{n\times k}\right|}$ [34].

**Proof** **of** **Lemma** **1.** As for matrix $B_{n\times k}$, where n > k, $B_{n\times k}=\left(\begin{array}{ccc} b_{11} & \ldots & b_{1k}\\ b_{21} & \ldots & b_{2k}\\ \vdots & \ddots & \vdots \\ b_{n1} & \ldots & b_{nk} \end{array}\right)=\left(b_{1},\ldots ,b_{k}\right)$. According to Gram Schmidt’s orthogonality theory, there is an orthogonal matrix ***U_n×k_*** and a lower triangular matrix ***C****_k×k_*. Hence, $B_{n\times k}$ is redrafted as follows:

(27) $$B_{n\times k}=U_{n\times k}C_{k\times k}$$

This is mathematically called QR decomposition. As we know, the orthogonal transformations do not change the geometric properties of matrices; the determinant of $B_{n\times k}$ is expressed as follows:

(28) $$\begin{array}{l} \det B_{n\times k}=\left|\det C_{k\times k}\right|=\sqrt{\det({C_{k\times k}}^{T}C_{k\times k})}\\ =\sqrt{\det[{\left({U_{n\times n}}^{T}B_{n\times k}\right)}^{T}({U_{n\times n}}^{T}B_{n\times k})]}=\sqrt{\det({B_{n\times k}}^{T}B_{n\times k})} \end{array}$$

Likewise, for matrix B with n×k and n>k, a reasonable definition of non-square determinant is $\det B_{n\times k}=\sqrt{\det(B_{n\times k}{B_{n\times k}}^{T})}$ when n<k. Hence, according to Lemma 1, Equation (26) is derived as follows:

(29) $$\left|\lambda \right|=\left|\Lambda \right|\times {\left(\left|Z_{t}\right|\times \left|Z_{\Delta t}\right|\right)}^{6}\times \left|Z_{t}\right|=\sqrt{\left|{\Lambda_{2\times 4}}^{T}\Lambda_{2\times 4}\right|}\times {\left(\left|Z_{t}\right|\times \left|Z_{\Delta t}\right|\right)}^{6}\times \left|Z_{t}\right|$$

**Figure 4:**
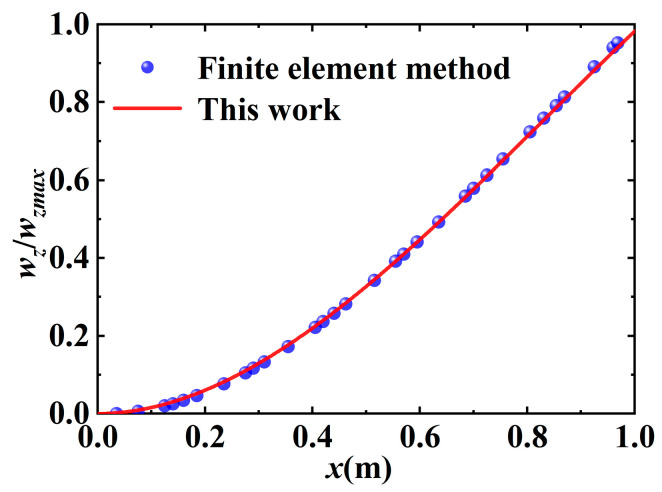
Comparison of first-order vibration mode obtained using mathematical model and finite element simulation.

**Figure 5:**
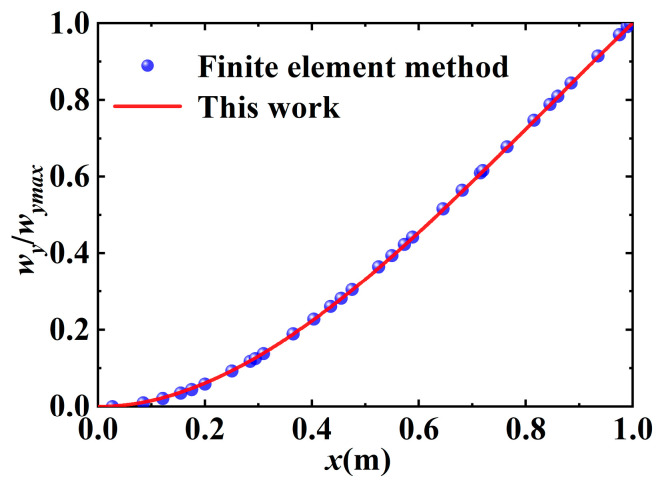
Comparison of second-order vibration mode obtained using mathematical model and finite element simulation.

By substituting Λ in Equation (18), $Z_{t}$ in Equation (22), and $Z_{\Delta t}$ in Equation (23) into Equation (26), Equation (29) can be expressed. In order for the equation to have a solution, the coefficient determinant of λ is 0. Hence, the transcendental equation for the natural frequency of a variable cross-section flexible cantilever beam can be obtained. Using Newton’s iterative method, the first-order natural frequency of the variable cross-section flexible cantilever beam can be obtained as 8.1261 Hz, and the second-order frequency is 17.721 Hz. By substituting the first two natural frequencies into Equation (7), the mode function of the variable cross-section flexible cantilever beam can be obtained. To simplify the solution, the mode function is fitted, and the simplified first two mode curves are shown in Figure 4 and Figure 5. The results indicate that the theoretical and simulation errors are minimal, and the overlap of the first two orders of vibration curves is up to 99%. The mode function can be used for subsequent research.

(30) $$\left\{\begin{array}{c} Y_{1}=\sin0.0564x-\sinh0.0564x-0.0472(\cos0.0564x-\cosh0.0564x)\\ Y_{2}=\sin0.0564x-\sinh0.0564x-0.0475(\cos0.0564x-\cosh0.0564x) \end{array}\right.$$

The cantilever beam is excited by displacement from the base, like $w_{Base}(0,t)=A\sin\omega_{Base}t$, and produces the forced vibration. Ignoring damping effects, Equation (1) is derived as follows:

(31) $$E_{b}I(x)\frac{\partial^{4}w\left(x,t\right)}{\partial x^{4}}+\rho S(x)\frac{\partial^{2}w\left(x,t\right)}{\partial t^{2}}=f(x,t)$$

After arithmetical transformations, the fully decoupled Equation (2) is derived as follows:

(32) $$q{\left(t\right)}^{\prime \prime }+{\omega_{j}}^{2}q\left(t\right)=Q_{j}(t),$$

where $Q_{j}(t)=\int_{0}^{l_{0}}f(x,t)Y_{j}(x)dx$. The general solution of Equation (32) based on Duhamel’s integral is the following:

(33) $$q_{j}(t)=\frac{1}{\omega_{j}}\int_{0}^{l}Q_{j}(\tau )\sin\omega_{j}(t-\tau )d\tau +q_{j}(0)\cos\omega_{j}t+\frac{{\dot{q}}_{j}(0)}{\omega_{j}}\sin\omega_{j}t$$

Making use of the initial condition, $Q_{j}(\tau )=0$, the forced vibration response of a cantilever beam under fixed-end excitation is expressed as follows:

(34) $$w(x,t)=\sum_{j=1}^{\infty }Y_{j}(x)q_{j}(t)=\sum_{j=1}^{\infty }Y_{j}(x)[\int_{0}^{l_{a}}\rho S(0)w_{Base}(x,0)Y_{j}dx\cos\omega_{j}t+\frac{\int_{0}^{l_{a}}\rho S(x){\dot{w}}_{Base}(x,0)Y_{j}dx}{\omega_{j}}\sin\omega_{j}t]$$

where $Y_{j}$ is the Simplification and Regularization model shape function. Due to the harmonic excitation of the base and its low frequency (less than 20 Hz), the proportion of higher-order harmonics is smaller. Equation (34) is described as follows:

(35) $$\varphi_{1}(x,t)=Y_{1}(x)q_{1}(t)=Y_{1}(x)\frac{\sin\omega_{1}t}{\omega_{1}}\int_{0}^{l_{a}}-\rho S(x)A\omega_{Base}\cos\omega_{Base}tY_{1}(x)dx$$

## 3. Parameters Analysis and Discussion

**Figure 6:**
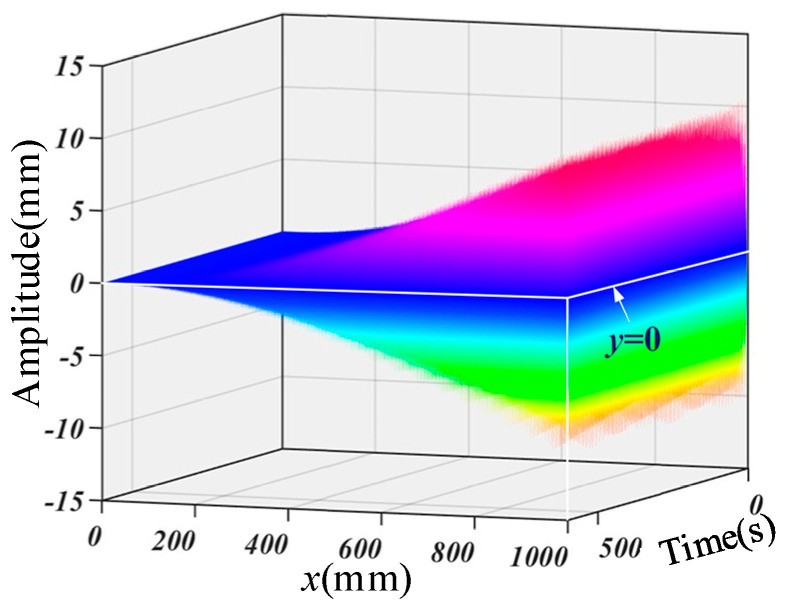
The response curve of the cantilever beam subjected to harmonic fixed-end excitation.

As mentioned in the previous section, the vibration curve and amplitude are associated with fixed-end excitation. The excited signal of the fixed-end of the cantilever beam is $w_{Base}(0,t)=5\sin8t$, which is the first-order natural frequency. According to Equation (35), the response curve of the variable cross-section homogeneous cantilever beam is shown in Figure 6. The vibration response is affected by coupled vibration via time. It is observed that under the excitation of the base, the curve is a variable amplitude approximately periodic curve, with a maximum amplitude of 10.38 mm, located at 26 s. Based on this, the subsequent response curve is guided.

### 3.1. Acquisition Point

**Figure 7:**
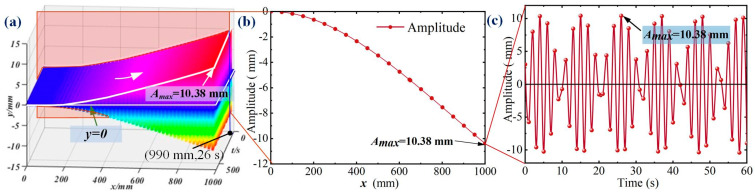
The relationship between vibration response and acquisition point position. (**a**) Response curves of vibration collected at intervals of 20 mm. (**b**) The response mode of the cantilever beam at 26 s. (**c**) The relationship between amplitude and time at the 990 mm acquisition point.

The vibration response curve is collected at intervals of 20 mm, as shown in Figure 7a. It indicates that the amplitude of the vibration response follows that the farther the distance from the base, the greater the amplitude. The response curve at 26 s is intercepted, as shown in Figure 7b. These figures indicate that the cantilever beam’s response vibration curve under fixed-end excitation is approximately quasi-static while separating the impact of response time. Figure 7c depicts the relationship between vibration response and time at a specific collection point on a cantilever beam. The fixed-end excitation has the coupled vibration effect on the response of the cantilever beam, resulting in periodic amplitude reduction.

### 3.2. Excitation Frequency

**Figure 8:**
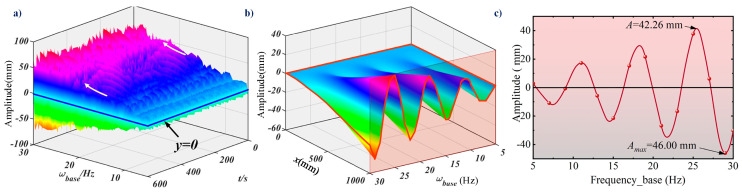
The relationship between vibration response and excitation frequency. (**a**) The response curve of the excitation frequency from 5~30 Hz at the 990 mm acquisition point. (**b**) The response mode of the cantilever beam at 26 s. (**c**) The relationship between vibration amplitude and excitation frequency at the 990 mm acquisition point.

As in the previous calculations, the responses of the cantilever beam were analyzed at the 990 mm acquisition point and 26 s. The influence of excitation frequency is shown in Figure 8. The excitation signal remained at the amplitude of 5 mm, and the excitation frequency range slided from 5 Hz to 30 Hz. In the case of the support motion, the output power of the base increases and the vibration energy of the cantilever increases. In this situation, the amplitude of the collection point (990 mm) increases with the excitation frequency, as shown in Figure 8a. There is a peculiar phenomenon in Figure 8b. When separating the effects of the collection time (The collection time is 26 s.), the amplitude oscillates for the cantilever beam. The significance of oscillation decreases as the distance from the fixed end decreases. At the 990 mm acquisition point, the relationship between amplitude and time is shown in Figure 8c. The oscillation forms a wave with gradually increasing amplitude up to 46 mm under 30 Hz. This phenomenon may be because the amplitude collected in this article is the vertical vibration of the cantilever beam. When the frequency exceeds its first-order natural frequency, there is a second-order and third-order equivalent coupled vibration, which attenuates in the vertical direction.

### 3.3. Excitation Amplitude

**Figure 9:**
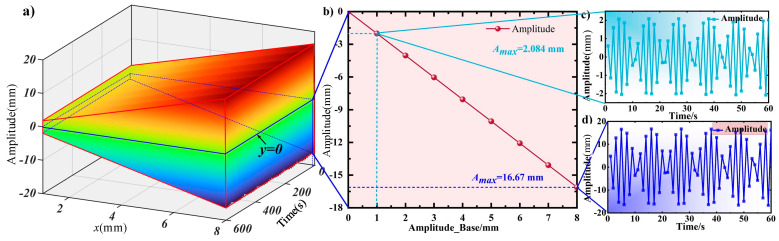
The relationship between response amplitude and excitation amplitude at the acquisition point 990 mm under 8 Hz. (**a**) The relationship between amplitude and driving amplitude. (**b**) The relationship between the amplitude of excitation and the response amplitude (**c**) The response over time under 1 mm excitation. (**d**) The response over time under 8 mm excitation.

The relationship between the response amplitude at the acquisition point and the driving amplitude under 8 Hz excitation is shown in Figure 9. Figure 9b shows that the maximum amplitude of the response at the acquisition point is linearly related to the driving voltage. The linear parameter obtained through least squares fitting is –2.084, expressed as $y=-2.084A_{base}$. The material and parameters of the cantilever beam itself influence the coefficient of fitting the curve. The response curve trends under different excitation amplitudes are similar, as shown in Figure 9c,d. These curves have the same frequency but different amplitudes. Combined with Figure 8, it can be concluded that the impact of excitation frequency on response amplitude is more significant than that of excitation amplitude. The change in excitation frequency will change the response mode shape, but the change in amplitude only affects the response amplitude.

## 4. Experimental Validation

**Figure 10:**
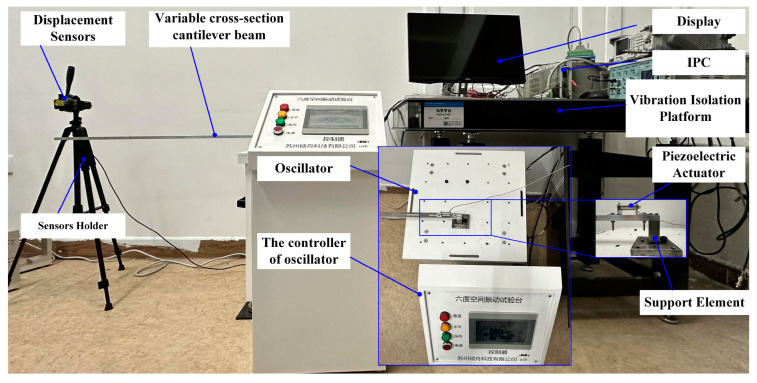
Experiment platform.

In this section, an experimental platform is constructed to validate the analysis of vibration characteristics in the stepwise cantilever beam, as shown in Figure 10. The platform allows us to investigate the response of the beam under various excitation amplitudes and frequencies and also to verify the influence of the collection point. The control system actuates the vibration table, inducing vibrations in the cantilever beam to simulate the vibration observed at the fixed end of a mechanical structure in practice. The displacement of the vibration is collected through the laser displacement sensor, and the amplitude is obtained through data processing. The filtering method employed in this study is low-pass filtering.

### 4.1. Acquisition Point in Experiment

In the experimental studies, the excitation frequency is 8 Hz, which is the first-order natural frequency of the cantilever beam. The excitation amplitude is 5 mm, which is the maximum excitation amplitude of the vibration table. The excitation signal is sine signal. Owing to the electromagnetic excitation of the vibration table, the signal cannot maintain a perfect sine curve under different frequency and amplitude.

**Figure 11:**
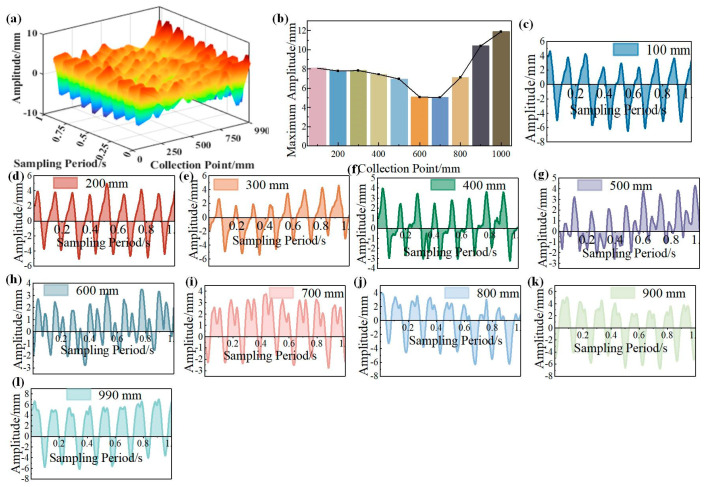
The relationship between vibration response and acquisition point position. (**a**) Response curves of vibration collected at intervals of 100 mm. (**b**) The response curves of vibration collected in the sampling period. (**c**) The response curve at 100 mm acquisition point. (**d**) Response curve at 200 mm acquisition point. (**e**) The response curve at 300 mm acquisition point. (**f**) The response curve at 400 mm acquisition point. (**g**) The response curve at 500 mm acquisition point. (**h**) The response curve at 600 mm acquisition point. (**i**) The response curve at 700 mm acquisition point. (**j**) The response curve at 800 mm acquisition point. (**k**) The response curve at 900 mm acquisition point. (**l**) The response curve at 990 mm acquisition point.

As shown in Figure 11a, the vibration curve in a sampling period almost maintained sinusoidal characteristics. Combined with Figure 11b, the maximum amplitude remains approximately unchanged within 400 mm, then suddenly decays and gradually increases. Notably, the maximum amplitude curve in Figure 11b is adjusted to account for the error caused by the movement of the vibration table, and the error is specifically reflected in the fact that the curves in Figure 11c–l are not symmetrical to line zero. Obviously, the asymmetry of the curves is also affected by the instability of the excitation signal. Figure 11c–l indicates the following:

(1) There are fluctuations in the curves in the amplitude direction. This signification illustrated that the vibration table has a movement in a direction perpendicular to the ground. This is owing to the vibration table not being fixed on the ground.

(2) The cantilever beam can be treated as rigid motion within 300 mm owing to the fact that the curves in Figure 11c–e kept almost identical mode shapes and amplitudes, which are familiar to the excitation signal.

(3) There is coupling between rigid body motion and vibration range from 400 mm to 700 mm. This coupled motivation led to the deflection of the amplitude, as shown in Figure 11f–h.

(4) The closer to the free end, the greater the amplitude when the cantilever beam is under forced vibration, as shown in Figure 11i–l.

The curve trend and amplitude are similar, but the experimental results are more disordered. This is because in the experiment, the exciter is not completely fixed, so it is difficult to ensure that the excitation signal received by the fixed end is completely consistent with that in the simulation. Besides that, the response of the cantilever beam is coupled with the rigid motion and continuous elastic body vibration.

### 4.2. Excitation Frequency in Experiment

**Figure 12:**
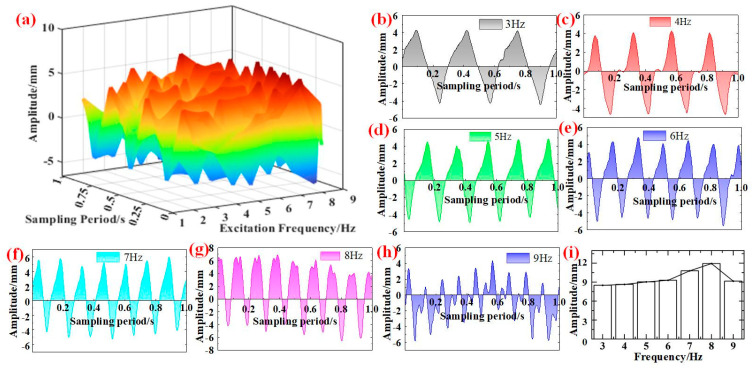
Different excitation frequency under 5 mm. (**a**) Relationship between vibration response and excitation frequency. (**b**) The response curve under 3 Hz. (**c**) The response curve under 4 Hz. (**d**) The response curve under 5 Hz. (**e**) The response curve under 6 Hz. (**f**) The response curve under 7 Hz. (**g**) The response curve under 8 Hz. (**h**) The response curve under 9 Hz. (**i**) Maximum amplitude under different excitation frequency.

Figure 12 illustrates the response curves of the cantilever beam at the 990 mm acquisition point under various excitation frequencies exhibiting an excitation amplitude of 5 mm. Owing to the vertical movement of the vibration table under different excitation frequency, Figure 12a shows that the irregular fluctuations that are specifically presented in the curves in Figure 12b–h are asymmetric to the x-axis. Separating the influence of the vibration table on the response curve, Figure 12i illustrates that the vibration amplitude increases with the increase in the excitation frequency. There is a deflection in Figure 12i under 9 Hz. The reason may be that the vibration table reaches the maximum output power, and the excitation amplitude is lower than 5 mm.

### 4.3. Excitation Amplitude in Experiment

**Figure 13:**
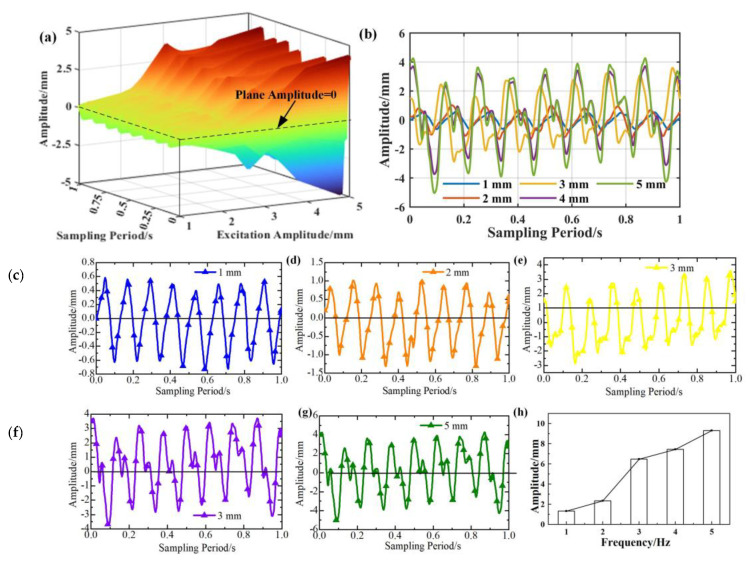
Different excitation amplitude under 8 Hz. (**a**) Relationship between vibration response and excitation frequency. (**b**) Response curves under different excitation amplitude. (**c**) Response curve under 1 mm. (**d**) The response curve under 2 mm. (**e**) The response curve under 3 mm. (**f**) The response curve under 4 mm. (**g**) The response curve under 5 mm. (**h**) The maximum amplitude under different excitation amplitude.

Figure 13a depicts the response curves of the cantilever beam under different excitation amplitudes while the fixed end oscillates at different frequencies. It can be concluded that the response curves are similar to the excitation signal and almost maintain the sine characteristics, as shown in Figure 13b. Figure 13h concentrates the maximum amplitude of the curves in Figure 13c–g. It illustrated that the amplitude of the response curves increased with the excitation amplitude. The experimental research has verified the results of theoretical derivation, as shown in Figure 9.

## 5. Direct Inverse Vibration Suppression Technology Based on Mathematical Model

### 5.1. Theoretical Analysis and Simulation

**Figure 14:**
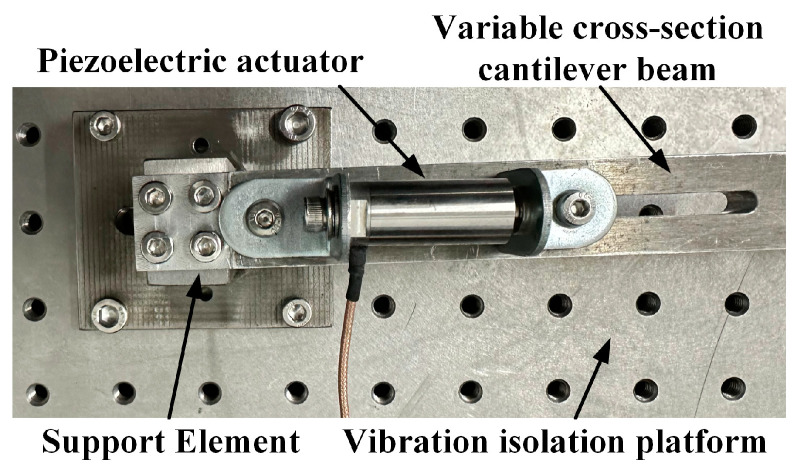
The physical diagram of piezoelectric actuator.

Based on the mathematical model of the forced vibration of the cantilever beam, this part designed a vibration suppression controller based on a piezoelectric actuator. The details of the actuator are shown in Figure 14, which is described in detail in Reference [35]. The piezoelectric actuator is installed at the fixed end. The mathematical model of the piezoelectric actuator driving the cantilever beam is the following:

(36) yact(t)=Y1(990)[Y˙1(ζ1)−Y˙1(ζ2)]α3(sinωactt)2(1−cosω1t)ω1[2p2ua(t)sin(ωactt)+ka]ua(t),

where *p*_2_ is the proportional coefficient of the piezoelectric actuator drive voltage, *u_a_* is the piezoelectric actuator drive voltage, $\omega_{act}$ is the piezoelectric actuator drive frequency, *k_a_* is the equivalent stiffness, $\alpha_{3}$ is the proportional coefficient, and $\xi_{1},\xi_{2}$ is the installation position on the cantilever beam.

The principle is that the response of the piezoelectric actuator driving the cantilever beam is equal in magnitude and opposite in direction to the forced vibration response of the cantilever beam at any given time, that is,

(37) $$\begin{array}{l} -y(t)=y_{act}(t)=-Y_{1}\frac{\rho A\omega_{base}\int_{0}^{l_{a}}S(x)Y_{1}dx}{\omega_{1}}\left[\sin\omega_{1}t-\omega_{base}\sin\omega_{base}t\right]\\ =\frac{\alpha_{3}}{p_{2}u_{a}(t)\sin(\omega_{1}t)+k_{a}}u_{a}(t)[\overset{\cdot }{Y_{1}(\zeta_{1})}-\overset{\cdot }{Y_{1}(\zeta_{2})}]\frac{\sin\omega_{1}t(1-\cos\omega_{1}t)}{\omega_{1}} \end{array}$$

Therefore, it can be inferred that the driving voltage of the piezoelectric actuator is the following:

(38) $$u_{a}(t)=\frac{Y_{1}\omega_{1}k_{a}\rho A\omega_{base}\int_{0}^{l_{a}}S(x)Y_{1}dx\left[\sin\omega_{1}t-\omega_{base}\sin\omega_{base}t\right]}{\omega_{1}\alpha_{3}[\overset{\cdot }{Y_{1}(\zeta_{1})}-\overset{\cdot }{Y_{1}(\zeta_{2})}]\sin\omega_{1}t(\cos\omega_{1}t-1)-Y_{1}\rho A\omega_{base}\int_{0}^{l_{a}}S(x)Y_{1}dx\left[\sin\omega_{1}t-\omega_{base}\sin\omega_{base}t\right]\omega_{1}p_{2}\sin\omega_{1}t}$$

**Figure 15:**
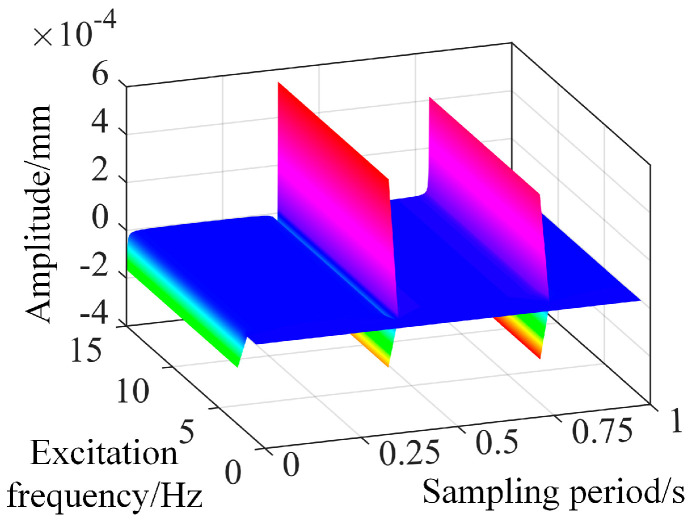
The simulation results of direct inverse control based on the vibration equation of the variable cross-section cantilever beam.

When the driving voltage frequency of the piezoelectric actuator matches the natural frequency of a cantilever beam, the response amplitude of the beam is at its maximum. During this scenario, the driving voltage is at its minimum, resulting in the lowest power loss. Consequently, the recommended driving frequency for the piezoelectric actuator is 8 Hz. By applying the voltage to the piezoelectric actuator using Equation (38) and simulating it, the displacement results of the cantilever beam at sampling points were obtained, as shown in Figure 15. The authors observed that within the sampling period, the controller achieves relatively good vibration suppression effects, indicating the basic correctness of the forced vibration dynamic model of the cantilever beam.

When the excitation frequency applied to the cantilever beam corresponds to its first-order natural frequency, $\omega_{1}=\omega_{base}$. The driving voltage can be derived as follows:

(39) ua(t)=Y1ω1ka2ρA∫0laS(x)Y1dx(1−ω1)sinω1tω1α3[Y1(ζ1)·−Y1(ζ2)·]sinω1t(cosω1t−1)−Y1ρAω1p22(1−ω1)(sinω1t)2∫0laS(x)Y1dx

**Figure 16:**
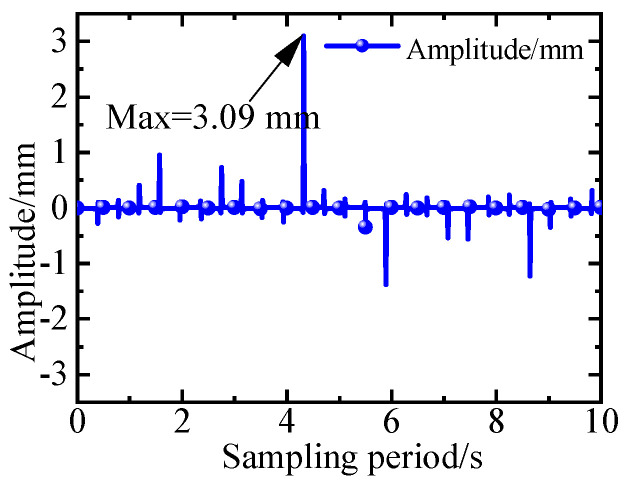
The simulation results from direct inverse control with 1^st^ order frequency.

According to the above equation, direct inverse control simulation reveals that the amplitude of the vibration after damping based on the direct inverse control system established on the vibrating equation of the variable-section cantilever beam, and the driving equation of the piezoelectric actuator can stabilize near 0 mm. However, within the sampling period, there is a sudden change point with a maximum amplitude of 3.09 mm as shown in Figure 16. It is observed that with an increase in sampling time, the number of sudden change points in the amplitude after damping increases, and the amplitude at these points also increases.

### 5.2. Experimental Validation for Direct Inverse Vibration Suppression Technology

**Figure 17:**
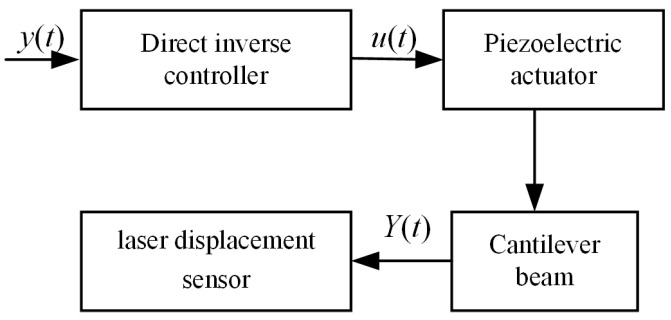
The block diagram of the direct inverse controller.

**Figure 18:**
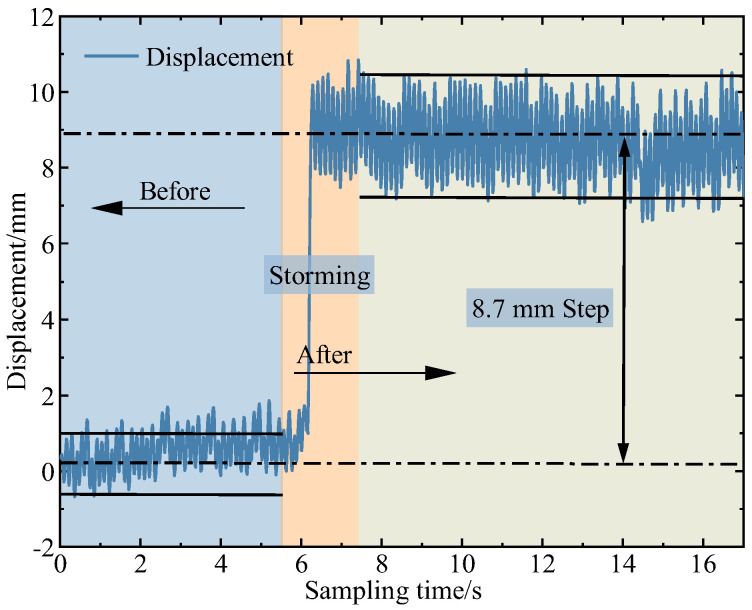
The ineffective vibration suppression experimental results of direct inverse control based on vibration equation.

**Figure 19:**
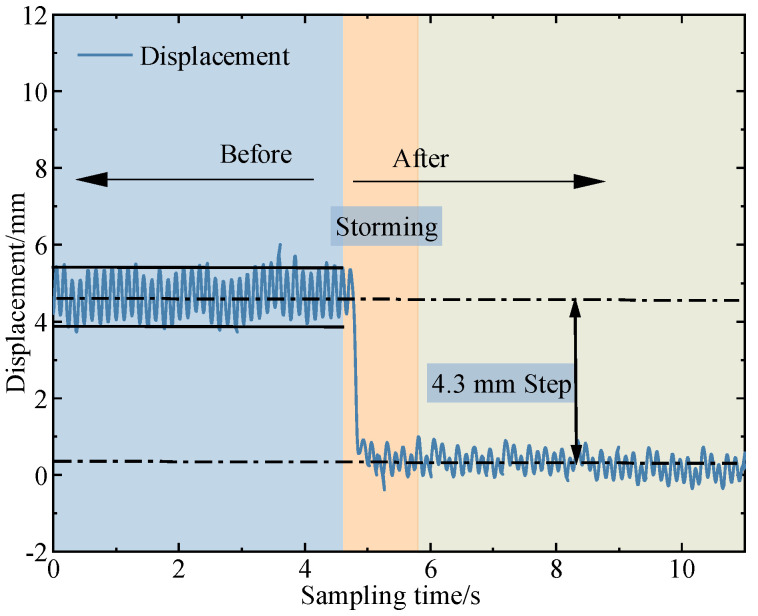
The effective vibration suppression experimental results of direct inverse control based on the vibration equation.

The experiment investigated the vibration suppression effect of the direct inverse controller based on the vibration equation. The block diagram of the direct inverse controller is shown in Figure 17. The experimental results are shown in Figure 18. The principle of the direct inverse controller based on the vibration equation does not involve feedback signals. It applies voltage to the piezoelectric actuator for vibration suppression at the moment of excitation, avoiding the use of displacement sensors. However, during the experiment, two diametrically opposite effects occur due to the instability of the excited signal. When there is an error between the excitation signal and the excitation signal in the dynamic model, the experiment shows an amplification effect in amplitude, as shown in Figure 19. When the excitation signal matches the excitation signal in the dynamic model, amplitude suppression can be achieved, with a suppression rate exceeding 50%. Two different experimental results show that although the direct inverse controller based on the vibration equation has a good vibration suppression effect in theoretical simulation, it is often affected by the phase factor of the vibration signal and vibration suppression signal in the experiment process, resulting in amplitude amplification. Therefore, the synchronization of vibration signals and vibration suppression signals should be studied in the future.

## 6. Conclusions

This paper focused on the end effector of substrate-handling robots. This study proposed the multi-stepwise beam model, built the mathematical model, and obtained the modal shape functions and the natural frequencies in the analytical solution. Meanwhile, the vibration response of the end effector under fixed-end excitation was analyzed based on the multi-step cantilever beam structure and proved through experimental research. The direct inverse vibration suppression controller verified the model’s accuracy while achieving 50% amplitude suppression.

The model takes complete account of the section variation and fixed-end attitude excitation. Meanwhile, this method reduces computational efforts by solving problems at the matrix level. The characteristic equation, modal frequencies, eigenfunction, and orthogonality conditions are obtained to circumvent traditional calculation methods’ errors. The overlap between numerical and simulation results is up to 99%, and the experimental research demonstrated the theoretical analysis results. This means that the method is accurate enough to supervise the design of subsequent accurate control.

Additionally, the mathematical analytic equation for the transient vibrations of the beam excited through base motion is derived from calculating transient beam responses during the passage through resonance. The results show that the response curve is coupled with the excitation and natural model shape, which is a periodic amplitude curve. Based on the vibration response equation derived from this method, a direct inverse control method is proposed for vibration suppression. The results show that when there is no phase difference between the driving and response signals, the vibration suppression rate is above 50%. This method can be promoted to the vibration solution for variable section beams. Subsequent research should improve the driver’s performance and explore the impact of phase difference on vibration suppression.

## Figures and Tables

**Table 1:** The variable cross-section flexible cantilever beam parameters.

Parameter	Symbol	Value
Length	*l*_0_	1000 mm
Width	*b*_0_	20 mm
Height	2*h*_0_	10 mm
Length of section transition segment	*l_t_*	105 mm
Interval length of mutation segment	δ*l_t_*	35 mm
Length of free end	*l_t_^’^*	55 mm
Density	*ρ*	2.7 g/cm^3^

## Data Availability

Data will be made available on request due to privacy.
